# Completion of ≥6 cycles of adjuvant temozolomide is the strongest modifiable prognostic factor in glioblastoma: a 17-year real-world cohort of 319 glioma patients from a single Chinese center

**DOI:** 10.3389/fonc.2026.1933534

**Published:** 2026-09-16

**Authors:** Pei Liu, Xiaotang Xiao, Beiquan Hu, Kebei Xu, Ting Liu, Xu Liu, Huixian Huang, Qiang Pang, Zhiping Lu, Defeng Qing, Yu Liang, Siting Lin, Hao Chen, Heming Lu

**Affiliations:** 1 First and Second Division of Department of Radiation Oncology, Guangxi Academy of Medical Sciences and People’s Hospital of Guangxi Zhuang Autonomous Region, Nanning, China; 2 Department of Neurosurgery, Guangxi Academy of Medical Sciences and People’s Hospital of Guangxi Zhuang Autonomous Region, Nanning, China

**Keywords:** *E*-value, glioblastoma, glioma, inverse probability weighting, real-world evidence, restricted mean survival time, survival analysis, temozolomide

## Abstract

**Background:**

Level-1 evidence supports adjuvant temozolomide (TMZ) after chemoradiotherapy in newly diagnosed glioblastoma, but many patients discontinue before completing the six cycles the regimen calls for—particularly in China, where long-term outcome data anchored to treatment intensity remain scarce. We quantified the independent prognostic value of completing ≥6 cycles of adjuvant TMZ in a 17-year single-center Chinese glioma cohort.

**Methods:**

We retrospectively assembled 319 patients with pathologically confirmed glioma treated between 2009 and June 2026 at a tertiary Chinese center; 264 with complete grade, follow-up, resection, and radiotherapy data (tier A) formed the primary cohort. Overall survival (OS) was analyzed by Kaplan–Meier estimation and grade-stratified Cox regression. Robustness of the TMZ ≥6-cycle effect was probed by landmark analyses at 3, 6, 9, and 12 months; inverse-probability-of-treatment weighting (IPTW); restricted mean survival time (RMST); cause-specific Cox regression; prespecified subgroup/interaction analyses; and *E*-value sensitivity analysis.

**Results:**

Median follow-up was 79 months (IQR 42–115); 133 patients (50.4%) died. Five-year OS was 81.8% in WHO I–II, 42.2% in III, and 24.2% in IV (log-rank *P* < 0.0001). The stratified Cox model showed that ≥6 adjuvant TMZ cycles independently halved mortality (adjusted HR 0.46, 95% CI 0.31–0.67, *P* < 0.001), alongside age ≥60 (HR 1.97, 1.30–2.98) and radiotherapy boost/SIB (HR 1.59, 1.01–2.49). The effect held across all four landmarks (HR 0.44–0.60), after IPTW (HR 0.42, 0.29–0.62), and in the cause-specific model (HR 0.45, 0.31–0.67). RMST yielded 17.0 additional months at 5 years and 30.3 months at 10 years within WHO IV. In histologic glioblastoma, the pattern was threshold-shaped: 1–5 cycles fared no better than none (HR 0.86, *P* = 0.62), whereas ≥6 cycles conferred a 64% mortality reduction (HR 0.36, 0.17–0.75). *E*-values were 3.79 (point estimate) and 2.33 (CI limit nearer the null).

**Conclusion:**

Completion of ≥6 cycles of adjuvant temozolomide was the strongest modifiable prognostic factor for OS in high-grade glioma, robust across multiple bias-mitigation frameworks, although residual confounding by unmeasured molecular subtype and baseline functional status cannot be fully excluded. Completion—not merely initiation—of adjuvant TMZ warrants explicit tracking as a quality-of-care metric.

## Introduction

On paper, the treatment path for a newly diagnosed glioblastoma patient is straightforward: maximal safe resection, concurrent chemoradiotherapy with temozolomide (TMZ), and six cycles of adjuvant TMZ—the Stupp regimen (1). In practice, the second and third steps of that path are far less uniform than the first. Hematologic toxicity forces dose reductions and delays; performance status declines with disease progression; financial and logistical burdens accumulate; and both patient and clinician are periodically asked whether the next cycle is worth the cost. Whether the patient finishes six cycles, stops at two, or never begins adjuvant TMZ at all is settled across many small conversations spread over half a year. The magnitude of survival benefit tied to completing that regimen—as opposed to merely initiating it—shapes each of those conversations. Two decades after the pivotal EORTC/NCIC trial, the Stupp regimen still anchors first-line therapy for newly diagnosed glioblastoma in every major guideline (1, 2), yet real-world completion of the six adjuvant cycles varies widely, and the incremental value of finishing versus stopping early has been documented predominantly in Western trials and cohort studies (3–5). Chinese real-world data are sparse, despite the country contributing an estimated 100,000 new glioma diagnoses each year (6), and the 2021 WHO CNS5 classification (7) has widened the gap between contemporary reporting standards and the historical Chinese cohorts, which lack systematic IDH, 1p/19q, and MGMT annotation. A Chinese neuro-oncologist counseling a patient about a fifth or sixth cycle of adjuvant TMZ therefore does so against a thinner evidence base than the guideline text implies. To address this gap, we assembled a 17-year single-center cohort of 319 glioma patients treated between January 2009 and June 2026 at a tertiary center in southern China, with granular per-patient records of surgery, radiotherapy dose and fractionation, TMZ cycles, targeted therapy, and salvage treatment. Because molecular annotation was not systematically available across the observation window, we deliberately anchored the analysis on modifiable treatment intensity—the aspect of care a clinician can actually change at the bedside—rather than on molecular subtyping, and prespecified completion of ≥6 cycles of adjuvant TMZ as the primary exposure. Retrospective evaluation of a treatment-intensity exposure raises three familiar threats—confounding by indication, immortal time bias, and non-proportional hazards over long follow-up—which we addressed with a convergent set of six analytic frameworks (stratified Cox regression, multi-timepoint landmark analysis, inverse-probability-of-treatment weighting, restricted mean survival time, cause-specific hazards, and *E*-value sensitivity analysis) rather than any single model. Against this background, our objectives were to (1) describe long-term overall survival in a Chinese real-world glioma cohort stratified by legacy WHO grade, (2) identify independent prognostic factors in a rigorous multivariable framework, and (3) quantify the survival benefit of completing ≥6 cycles of adjuvant TMZ and test whether that benefit withstands the biases that most often undermine treatment-intensity claims in retrospective data.

## Methods

### Study design and cohort assembly

We conducted a retrospective observational cohort study reported in accordance with the STROBE guideline. All patients with a pathologically confirmed diagnosis of glioma (any grade) treated at Guangxi Academy of Medical Sciences and People’s Hospital of Guangxi Zhuang Autonomous Region between January 2009 and June 2026 were eligible. Data were extracted from a prospectively maintained institutional neuro-oncology registry, supplemented by chart review and structured telephone follow-up (median 3.4 contacts per living patient). The study was approved by the Institutional Review Board of Guangxi Academy of Medical Sciences and People’s Hospital of Guangxi Zhuang Autonomous Region. A waiver of written informed consent was granted for the retrospective extraction.

### Data cutoff and censoring

A single database lock date of 30 June 2026 was applied to all analyses; information obtained after that date, whether new follow-up contacts or death notifications, was not included in the present report. For living or lost patients, observation time was defined as the interval from the date of pathological diagnosis to the last documented contact on or before the cutoff. For decedents, observation time was defined as the interval from diagnosis to date of death. Six patients had a documented month and year of death but no day; these were imputed to the 15th of the month, and a sensitivity analysis confirmed no material impact.

### Data cleaning and variable derivation

The registry comprised 66 raw fields per patient, mixing coded, semistructured, and free-text entries. Free-text fields were transformed by rule-based parsing into standardized variables using a reproducible Python pipeline. Key derivations were as follows: Radiotherapy dose was decomposed from free-text prescriptions (e.g., “PTV1–60 Gy in 30 fx + PTV2 boost 6 Gy”) into six structured fields—total dose (Gy), total fractions, dose per fraction, primary-phase dose and fractions, boost dose and fractions, and boost presence (binary)—with an accompanying parse type [single-phase, sequential, simultaneous integrated boost (SIB), multiphase, or none]; adjuvant TMZ cycles were recorded as an integer, with the primary exposure defined as ≥6 vs. <6 cycles (the latter including patients who received no adjuvant TMZ at all); WHO grade was recorded as reported at the time of pathological diagnosis under the then-current WHO CNS classification (2007 for cases before 2016, the 2016 revision for 2016–2020, and the 2021 CNS5 for 2021 onward). No retrospective molecular reclassification was performed; the diagnostic edition was retained as a covariate. For analytic parsimony, grade I (*n* = 6, all pilocytic or pilomyxoid astrocytoma) was combined with grade II into a single “I–II” stratum; extent of resection (EOR) was determined from the operating surgeon’s report together with early postoperative imaging where available. A strict 48–72-h postoperative MRI was not obtained in every patient—particularly among earlier-era cases managed under legacy record systems—and where imaging within that window was unavailable, EOR was classified from the operative and discharge documentation. EOR was classifiable in 285 of 319 patients (89.3%); the 34 patients (10.7%) in whom it could not be classified were assigned to an “Unknown” category and, by construction, fell outside the tier A analytic cohort (which required a classifiable EOR). Early postoperative MRI within the 48–72-h window was more consistently available for patients treated in later years. EOR was recorded as gross total, presumed complete (no residual), subtotal, partial, or biopsy only. For multivariable analysis, EOR was dichotomized as gross total resection (gross total or presumed complete/no residual on the postoperative scan; *n* = 229) versus non-gross total resection (subtotal, partial, or biopsy-only; *n* = 35). Categorical reading rather than volumetric quantification of residual enhancing tumor is discussed as a limitation in the *Discussion*; recurrence was defined as any imaging (MRI or CT) or histological evidence of tumor recurrence or progression at any post-treatment timepoint. Cause of death was classified from family-reported information (obtained by telephone follow-up) or medical record if patients died in hospital as tumor-related, infection, cardio- or cerebrovascular, other, or unknown; no autopsy confirmation was available. In this cohort, “targeted therapy” refers exclusively to anti-angiogenic and anti-EGFR agents used at recurrence or for refractory disease (bevacizumab, anlotinib, apatinib, endostatin, and nimotuzumab); no patient received vorasidenib or any other IDH inhibitor, which was not approved in China during the accrual and treatment window (2009–June 2026).

### Data-quality tiers

For patient records that were incomplete with respect to one or more key fields, rather than deleting them, we implemented a three-tier data-quality classification and reported the analytic *n* at each stage. Tier A (*n* = 264) comprised patients with complete data on all key fields, namely, WHO grade, diagnosis date, survival status, overall survival, extent of resection, and radiotherapy dose. Tier B (*n* = 28) consisted of patients whose primary outcome was complete but who had non-critical incomplete data on extent of resection or radiotherapy dose. Tier C (*n* = 27) captured the remaining patients, each missing at least one key field (WHO grade, overall survival, diagnosis date, or survival status). Tier A formed the primary analysis cohort; tiers B and C are shown in the study flow diagram (**Figure 1**) and used in a prespecified sensitivity analysis. Per-variable completeness across all 42 study variables, in both the full registered cohort (*n* = 319) and the tier A cohort (*n* = 264), is reported in **Supplementary Table 1**.

**Figure 1:**
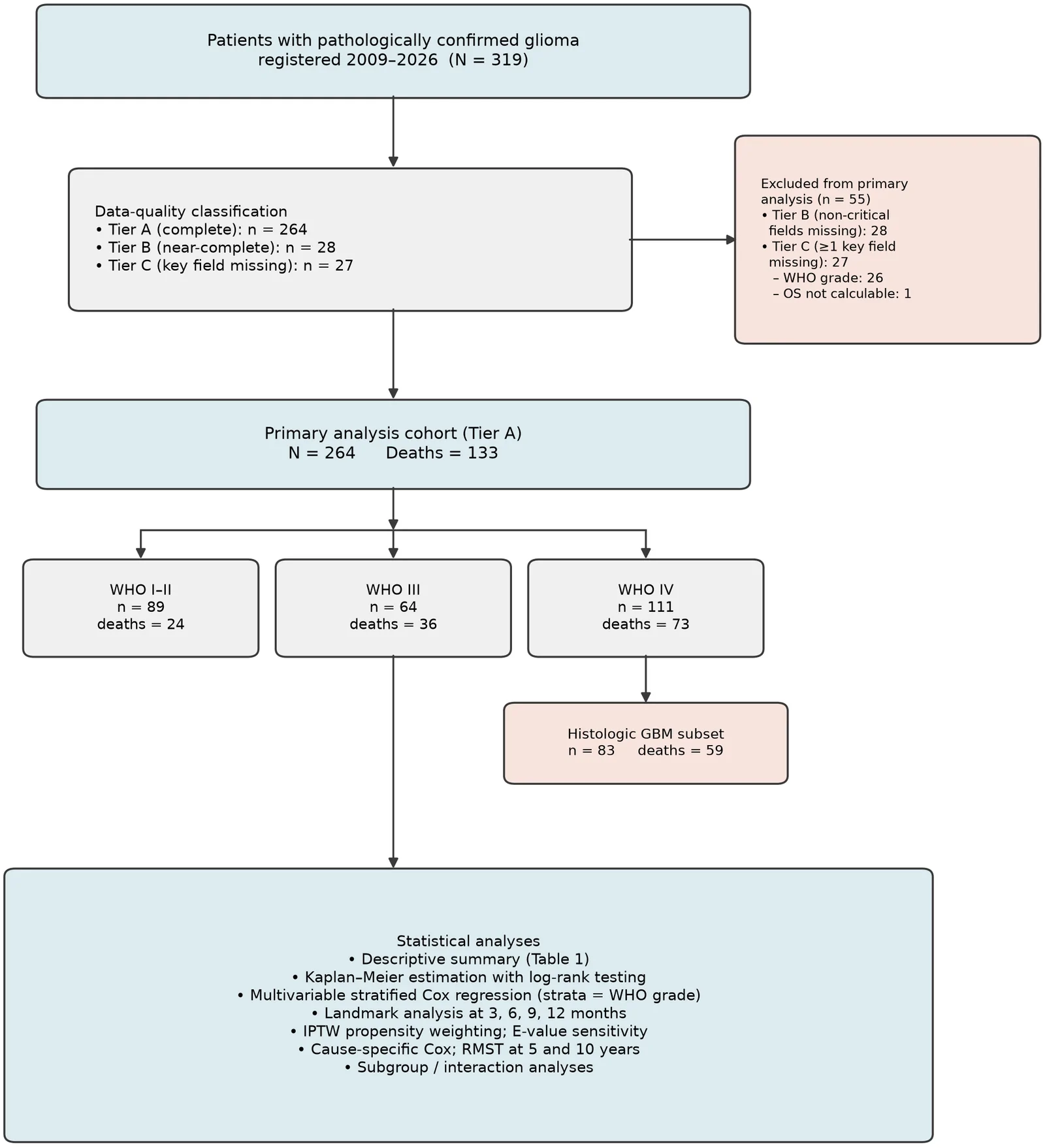
Patient selection and analytic flow diagram.

### Statistical analysis

The primary endpoint was overall survival (OS), defined as the time from the date of diagnosis to death from any cause. Patients alive at the cutoff or lost to follow-up were censored at their last documented contact. Continuous variables were summarized as median (interquartile range) and categorical variables as *n* (%), overall and stratified by WHO grade (**Table 1**). Cumulative survival was estimated by the Kaplan–Meier method with 95% confidence intervals; groups were compared by log-rank test, and number-at-risk tables accompany each Kaplan–Meier plot. For multivariable modeling, the primary model was a Cox proportional hazards regression stratified on WHO grade (I–II/III/IV) to accommodate non-proportional hazards, with prespecified covariates: age ≥60 years, male sex, non-gross total resection, no radiotherapy, radiotherapy boost or SIB, completion of ≥6 cycles of adjuvant TMZ, and receipt of targeted therapy. Effects were reported as hazard ratios (HRs) with 95% confidence intervals and Wald *P*-values. The proportional hazards assumption was assessed by the Schoenfeld residual test and complementary log–log survival plots. Covariates violating proportionality were handled either by stratification (WHO grade) or by additional time-split analyses; for those whose Schoenfeld test rejected the assumption, we additionally fit a time-split Cox model at a 24-month cutpoint and reported period-specific HRs. To rule out immortal time bias for the TMZ ≥6-cycle exposure, we refit the multivariable stratified Cox model at four landmark timepoints (3, 6, 9, and 12 months), including only patients still alive and event-free at each landmark and re-anchoring the time origin to zero. To further address confounding by indication, we performed inverse probability of treatment weighting (IPTW): propensity scores for adjuvant TMZ ≥6 cycles were estimated by logistic regression on age (continuous), sex, non-GTR, radiotherapy boost/SIB, and WHO grade (dummy-coded), restricted to patients who received radiotherapy; stabilized weights were trimmed at the 1st and 99th percentiles, and covariate balance was assessed by standardized mean differences before and after weighting. The primary Cox model was then refit with the weights, using robust standard errors. Restricted mean survival time (RMST) was calculated at 60 and 120 months as the area under the Kaplan–Meier curve, with 95% confidence intervals from 500-replicate bootstrap resampling; between-group differences (ΔRMST) were tested with a normal approximation *P*-value. RMST was chosen as a hazard-ratio-independent effect measure well suited to comparisons in which the proportional hazards assumption is violated. Non-parametric cumulative incidence functions were estimated by the Aalen–Johansen method, with tumor-related death (including cause unknown, conservatively attributed to tumor) and non-tumor death as competing events; a cause-specific stratified Cox regression was fit for each cause. To probe heterogeneity of the TMZ ≥6-cycle effect, we performed prespecified subgroup analyses across 15 strata and formally tested for effect modification by WHO grade and by age via likelihood ratio comparison of nested Cox models. The *E*-value for the primary TMZ HR—and for the CI limit closer to the null—was computed with the VanderWeele–Ding formula; it quantifies the minimum joint strength of association (with both the exposure and the outcome) that an unmeasured confounder would need to have, above and beyond the measured covariates, to explain the observed effect away entirely. Sensitivity analyses comprised (1) recoding all lost-to-follow-up patients as deaths (worst-case scenario); (2) extending the analytic cohort to include tier B and C patients; and (3) excluding the six patients with imputed day of death. Three further sensitivity analyses of the TMZ ≥6-cycle effect were added during revision in response to reviewer requests: (4) excluding all recipients of targeted therapy; (5) restricting to patients with documented IDH status, with and without IDH mutation status as a covariate; and (6) adding baseline pre-radiotherapy performance status (PS ≥2 vs. <2) as a covariate in the subset with cleanly coded PS. All analyses were conducted in Python 3.11 using pandas 2.x, lifelines 0.28, matplotlib 3.x, and SciPy 1.x. A two-sided *P* < 0.05 was considered statistically significant, without correction for multiple comparisons—the primary analysis was confirmatory and the subgroup analyses were explicitly exploratory.

**Table 1:** Baseline and treatment characteristics of the primary analysis cohort, stratified by WHO grade.

Variable	Overall (Tier A)	I–II	III	IV
n (%)	264 (100.0)	89 (100.0)	64 (100.0)	111 (100.0)
Age, median (IQR)	47 (35–57)	39 (29–48)	47 (34–56)	53 (44–62)
Age ≥60, n (%)	52 (19.7)	8 (9.0)	12 (18.8)	32 (28.8)
Sex, male, n (%)	167 (63.3)	56 (62.9)	40 (62.5)	71 (64.0)
Histology				
Glioblastoma	83 (31.4)	—	—	83 (74.8)
Anaplastic astrocytoma	21 (8.0)	—	21 (32.8)	—
Astrocytoma	43 (16.3)	37 (41.6)	6 (9.4)	—
Oligodendroglioma	24 (9.1)	14 (15.7)	10 (15.6)	—
Oligoastrocytoma	18 (6.8)	13 (14.6)	5 (7.8)	—
Gliosarcoma	3 (1.1)	—	—	3 (2.7)
Pilocytic/PMA	2 (0.8)	2 (2.2)	—	—
Glioma NOS	45 (17.0)	16 (18.0)	11 (17.2)	18 (16.2)
Other/Unknown	9 (3.4)	3 (3.4)	4 (6.2)	2 (1.8)
Extent of resection				
Gross total	224 (84.8)	78 (87.6)	54 (84.4)	92 (82.9)
Presumed complete (no residual)	5 (1.9)	1 (1.1)	1 (1.6)	3 (2.7)
Subtotal	3 (1.1)	1 (1.1)	—	2 (1.8)
Partial	29 (11.0)	8 (9.0)	8 (12.5)	13 (11.7)
Biopsy only	3 (1.1)	1 (1.1)	1 (1.6)	1 (0.9)
Tumor maximum dimension, mm, median (IQR)	52 (40–64)	53 (37–64)	53 (43–66)	51 (41–62)
Radiotherapy				
Received radiotherapy, n (%)	240 (90.9)	76 (85.4)	64 (100.0)	100 (90.1)
Total dose, Gy, median (IQR)	60.0 (52.2–60.0)	54.0 (50.0–54.0)	60.0 (54.8–60.0)	60.0 (59.8–60.0)
Dose per fraction, Gy, median	2.00	2.00	2.00	2.00
Boost or SIB, n (% of RT recipients)	148 (61.7)	29 (38.2)	47 (73.4)	72 (72.0)
Systemic therapy				
Concurrent TMZ, n (%)	214 (81.1)	59 (66.3)	55 (85.9)	100 (90.1)
Adjuvant TMZ (any), n (%)	141 (53.4)	50 (56.2)	35 (54.7)	56 (50.5)
Adjuvant TMZ ≥6 cycles, n (%)	108 (40.9)	41 (46.1)	27 (42.2)	40 (36.0)
Stupp-like regimen, n (%)	129 (48.9)	43 (48.3)	33 (51.6)	53 (47.7)
Targeted therapy, n (%)	45 (17.0)	1 (1.1)	8 (12.5)	36 (32.4)

EOR, extent of resection; IQR, interquartile range; NOS, not otherwise specified; PMA, pilomyxoid astrocytoma; RT, radiotherapy; SIB, simultaneous integrated boost; TMZ, temozolomide.

Percentages are calculated with the column n as denominator, except for "Boost or SIB" for which the denominator is the number of radiotherapy recipients within the column. WHO grade I (n = 6, all pilocytic or pilomyxoid astrocytoma) was combined with grade II into a single "I–II" stratum for analytic parsimony, consistent with the multivariable Cox model. "—" indicates that no patients in the stratum fell into the category. Outcome variables (deaths, median OS, median PFS) are reported in the main text (the "Cohort composition and analytic flow" section–the "Overall survival by WHO grade" section) and **Figures 2**, **3**, and **6**. For the multivariable model, gross total and presumed complete (no residual) resections were combined as gross-total resection (GTR; n = 229, 86.7%); subtotal, partial, and biopsy-only resections formed the non-GTR group (n = 35).

## Results

### Cohort composition and analytic flow

Of 319 patients with pathologically confirmed glioma registered between January 2009 and June 2026, 264 (82.8%) met tier A criteria and formed the primary analysis cohort (**Figure 1**). Fifty-five patients were excluded: 26 had missing WHO grade, 1 had no calculable OS, and the remainder lacked one or more tier A fields. Median age at diagnosis was 47 years (IQR 35–57); 167 patients (63.3%) were male (**Table 1**). WHO grade was I–II in 89 patients (33.7%), III in 64 (24.2%), and IV in 111 (42.0%), of whom 83 (74.8% of grade IV) had histologic glioblastoma. Gross total resection was achieved in 224 patients; a further 5 had no residual tumor on the early postoperative scan, giving 229 (86.7%) classified as gross total resection for analysis. Radiotherapy was delivered to 240 patients (90.9%), with a median prescribed total dose of 60 Gy (IQR 52–60) at 2 Gy per fraction; 148 (61.7% of RT recipients) received a radiotherapy boost or SIB. Concurrent TMZ was given to 214 patients (81.1%), adjuvant TMZ to 141 (53.4%), and ≥6 cycles of adjuvant TMZ were completed by 108 (40.9%). A Stupp-like regimen (concurrent plus any adjuvant TMZ) was administered to 129 patients (48.9%). Targeted therapy was received by 45 patients (17.0%), concentrated in grade IV (32.4%). Median follow-up in the analytic cohort, estimated by the reverse Kaplan–Meier method, was 79 months (IQR 42–115), reaching a maximum of 183 months. At database lock, 133 patients (50.4%) had died, and 34 (10.7% of the original 319) were lost to follow-up.

### Overall survival by WHO grade

Grade-stratified survival showed the expected steep gradient (**Figure 2**, log-rank *P* < 0.0001). Median OS was not reached in WHO I–II, was 36.0 months in WHO III, and was 22.2 months in WHO IV. Five- and 10-year OS were 81.8% and 64.9% in WHO I–II, 42.2% and 28.7% in WHO III, and 24.2% and 19.0% in WHO IV, respectively.

**Figure 2:**
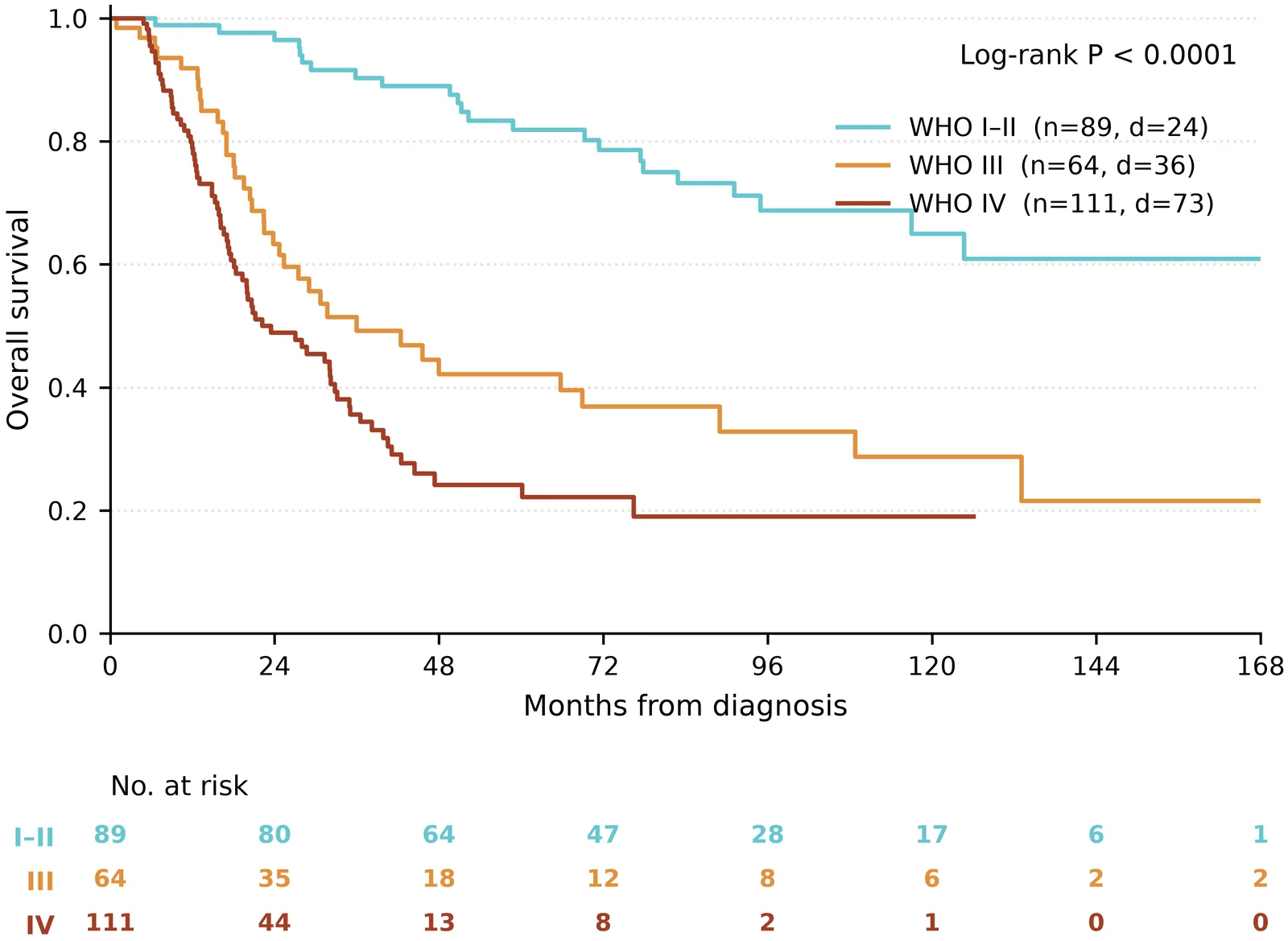
Overall survival by WHO grade in the tier A cohort (*n* = 264), Kaplan–Meier estimation with a number-at-risk table.

### Multivariable prognostic factors (primary model)

Before adjustment, Kaplan–Meier curves stratified by adjuvant TMZ exposure showed a marked survival advantage for patients completing ≥6 cycles (median OS not reached vs. 34.9 months for <6 cycles; log-rank *P* < 0.001; **Figure 3A**). To quantify this while controlling for grade and other prognostic factors, we fit a stratified multivariable Cox regression (strata = WHO grade; *n* = 264, 133 events; **Figure 4**, **Table 2**), which identified one modifiable protective factor and two independent adverse covariates: (1) completion of ≥6 cycles of adjuvant TMZ: adjusted HR 0.46 (95% CI: 0.31–0.67), *P* < 0.001; (2) age ≥60 vs. <60 years: adjusted HR 1.97 (95% CI: 1.30–2.98), *P* = 0.001; and (3) radiotherapy boost or SIB vs. none: adjusted HR 1.59 (95% CI: 1.01–2.49), *P* = 0.044. Non-significant covariates included male sex (HR 1.22), non-GTR resection (HR 1.04, essentially null), no radiotherapy (HR 1.46, wide CI), and targeted therapy (HR 1.45, upper CI 2.36). We interpret the RT-boost signal as confounding by indication—higher-grade tumors more frequently received boost or SIB (see the *Discussion* section below)—rather than as a causal detriment of the boost technique itself. Median age rose with grade (WHO I–II 39, III 47, IV 53 years; **Table 1**); because the primary Cox model is stratified on WHO grade, the age ≥60 hazard ratio is a within-grade estimate—comparing older with younger patients in the same grade stratum—and not a proxy for the grade effect.

**Figure 3:**
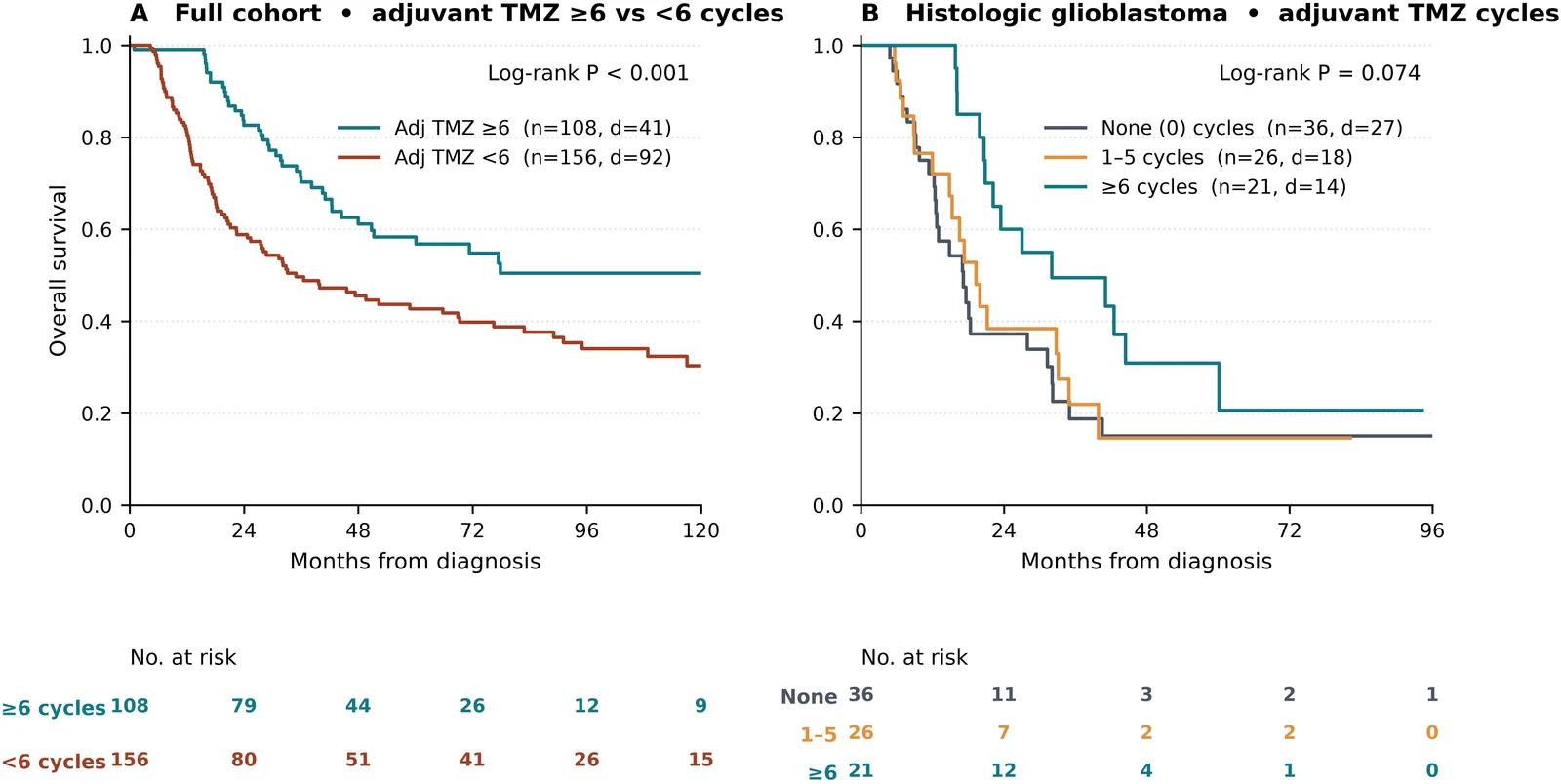
Overall survival by adjuvant temozolomide exposure. **(A)** Full cohort, adjuvant TMZ ≥6 vs. <6 cycles. **(B)** Histologic glioblastoma, adjuvant TMZ 0/1–5/≥6 cycles.

**Figure 4:**
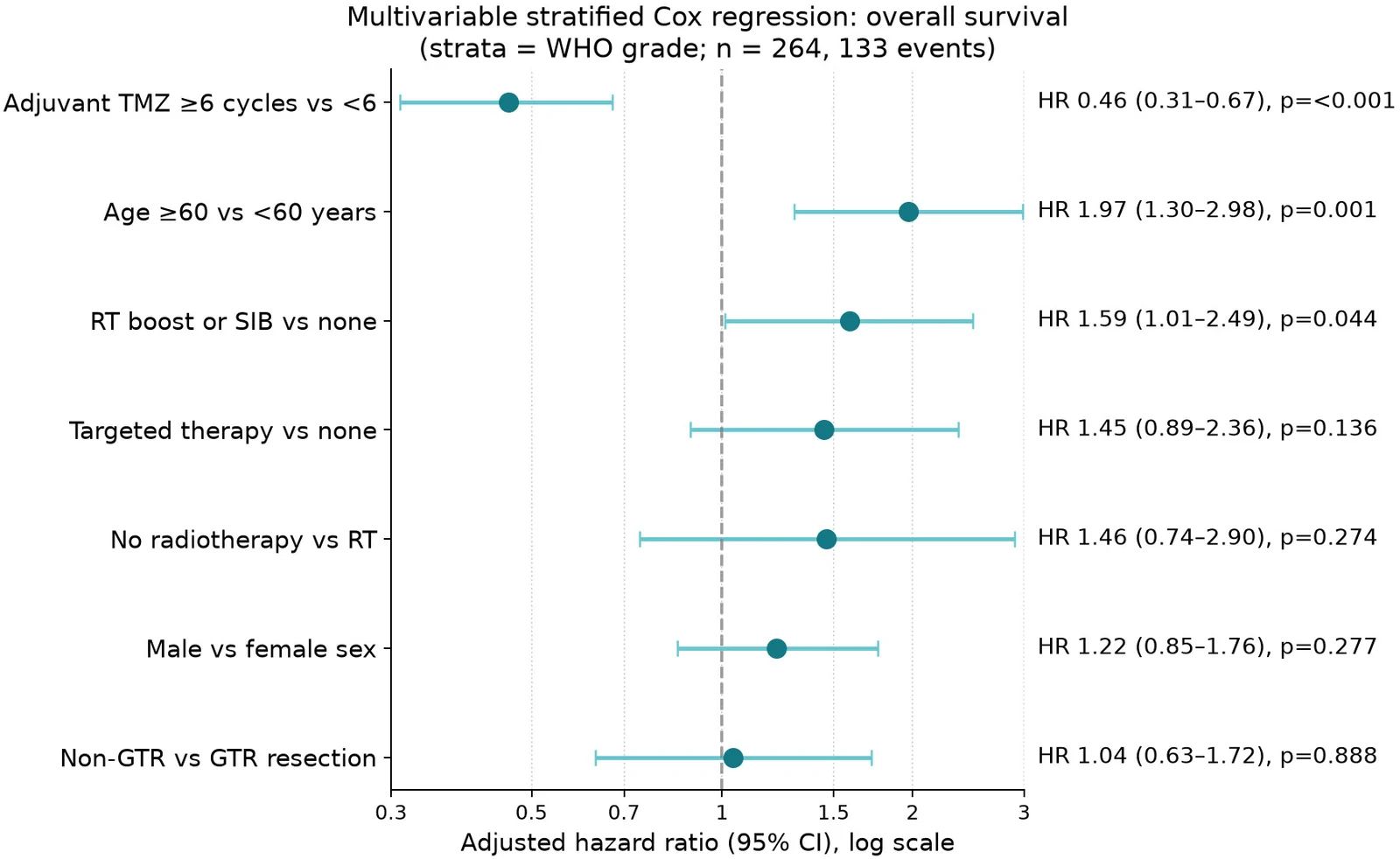
Multivariable stratified Cox regression (strata = WHO grade; *n* = 264), primary model.

**Table 2:** Multivariable stratified Cox regression primary model.

Covariate	HR	95% CI	P value
Adjuvant TMZ ≥6 cycles vs <6	0.46	0.31–0.67	<0.001
Age ≥60 vs <60 years	1.97	1.30–2.98	0.001
Radiotherapy boost or SIB vs none	1.59	1.01–2.49	0.044
Targeted therapy vs none	1.45	0.89–2.36	0.136
No radiotherapy vs radiotherapy	1.46	0.74–2.90	0.274
Male vs female sex	1.22	0.85–1.76	0.277
Non-gross-total vs gross-total resection	1.04	0.63–1.72	0.888

CI, confidence interval; HR, hazard ratio; SIB, simultaneous integrated boost; TMZ, temozolomide.

Cox proportional-hazards model stratified on WHO grade (I–II / III / IV) to accommodate non-proportional hazards for grade (Schoenfeld residual test P = 0.019 for grade IV, P = 0.035 for grade III). All seven covariates were prespecified and entered simultaneously. Covariates are ordered by P value ascending to place statistically significant effects at the top. Two-sided Wald P values are reported without multiplicity correction; the primary analysis is confirmatory.

### Non-proportional hazards and time-split analysis

Schoenfeld residual testing rejected the proportional hazards assumption for WHO grade IV (*P* = 0.019), WHO grade III (*P* = 0.035), adjuvant TMZ ≥6 (*P* = 0.002), and receipt of radiotherapy (*P* = 0.023). Stratifying on WHO grade in the primary model resolved the grade-related violation. For the remaining covariates, a time-split Cox model at a 24-month cutpoint showed that the TMZ ≥6-cycle benefit was concentrated in the first 24 months from diagnosis (HR 0.29, 95% CI: 0.17–0.51, *P* < 0.001) and attenuated to a non-significant HR 0.83 (0.46–1.47) beyond 24 months—consistent with a biological pattern of “clearing an early danger window” rather than a persistent multiplicative effect.

### Landmark analyses (immortal time bias)

Because completing ≥6 cycles of adjuvant TMZ typically requires a patient to survive approximately 6 months beyond radiotherapy, immortal time bias is an intrinsic concern for any naive comparison. Landmark analyses at 3, 6, 9, and 12 months from diagnosis (**Supplementary Table 2**) yielded consistent adjusted HRs of 0.44 (95% CI: 0.30–0.65), 0.47 (0.32–0.70), 0.54 (0.36–0.82), and 0.60 (0.40–0.92), respectively—all statistically significant. The effect is therefore not an artifact of the survival time required to accumulate exposure.

### Inverse probability of treatment weighting

Among the 240 radiotherapy-treated patients eligible for the propensity model, preweighting standardized mean differences reached 0.18 for both non-GTR and WHO grade IV. After stabilized inverse probability weighting, every covariate SMD fell below 0.01, indicating excellent balance. The IPTW-weighted stratified Cox model estimated an adjusted HR of 0.42 (95% CI: 0.29–0.62), *P* < 0.001 for adjuvant TMZ ≥6 cycles—nearly identical to the unweighted primary model, suggesting the association is not driven by measured confounding.

### Restricted mean survival time

RMST provided an HR-independent view of the effect (**Supplementary Table S3**). Within WHO grade IV, patients completing ≥6 cycles of adjuvant TMZ gained a mean of 17.0 additional months of survival over 5 years (ΔRMST 17.0, 95% CI: 9.2–24.7, *P* < 0.001) and 30.3 additional months over 10 years (95% CI: 11.8–48.8, *P* = 0.001), compared with patients completing fewer than 6 cycles. The corresponding differences within WHO grade III were in the same direction but did not reach significance (ΔRMST 9.3 months at 5 years, *P* = 0.08).

### Glioblastoma-specific analysis: a threshold effect of TMZ cycle count

Among the 83 histologic glioblastomas, a three-way categorization of adjuvant TMZ (none, *n* = 36; 1–5 cycles, *n* = 26; ≥6 cycles, *n* = 21) revealed a threshold rather than a dose–response pattern (**Figure 3B**). Median OS was 17.2, 19.3, and 32.0 months, respectively. In a Cox model with the same covariates as the primary model, 1–5 cycles did not differ significantly from no adjuvant TMZ (HR 0.86, 95% CI: 0.46–1.59, *P* = 0.62), whereas ≥6 cycles conferred a 64% reduction in mortality (HR 0.36, 95% CI: 0.17–0.75, *P* = 0.006). The three-way log-rank test did not reach statistical significance (*P* = 0.074), reflecting the close overlap of the 0- and 1–5-cycle curves. Among glioblastoma patients who initiated adjuvant TMZ but received only 1–5 cycles (*n* = 26), documented tumor progression was recorded in 16 (62%), indicating that early progression—rather than elective discontinuation—is the dominant reason patients did not reach 6 cycles. Median PFS mirrored the OS threshold pattern: 9.2 months with no adjuvant TMZ (*n* = 36), 10.2 months with 1–5 cycles (*n* = 26), and 16.8 months with ≥6 cycles (*n* = 21); the 0- and 1–5-cycle groups were essentially superimposed on PFS, whereas ≥6 cycles stood clearly apart.

### Subgroup and interaction analyses

Prespecified subgroup analyses (**Figure 5**, **Supplementary Table 4**) showed that the TMZ ≥6-cycle benefit was consistent across strata of age, sex, and extent of resection, with the largest effect in histologic glioblastoma (HR 0.33, 95% CI: 0.18–0.60). Two subgroups yielded point estimates above 1.0. The first was WHO I–II (HR 1.33, 95% CI 0.57–3.08), where adjuvant TMZ is not part of standard practice and the indication for its use is inherently different. The second was the earliest diagnosis era, Dx 2009–2015 (HR 1.28, 95% CI: 0.64–2.53, *P* = 0.486), which predates the period during which consistent 6-cycle prescribing became routine at our center; the estimate did not reach statistical significance, and later eras showed the expected protective direction (Dx 2016–2020 HR 0.53; Dx 2021–2026 HR 0.16). A formal test for TMZ × WHO grade interaction was statistically significant (likelihood-ratio *χ*² = 6.06, *df* = 2, *P* = 0.048), whereas TMZ × age showed no interaction (*P* = 0.82).

**Figure 5:**
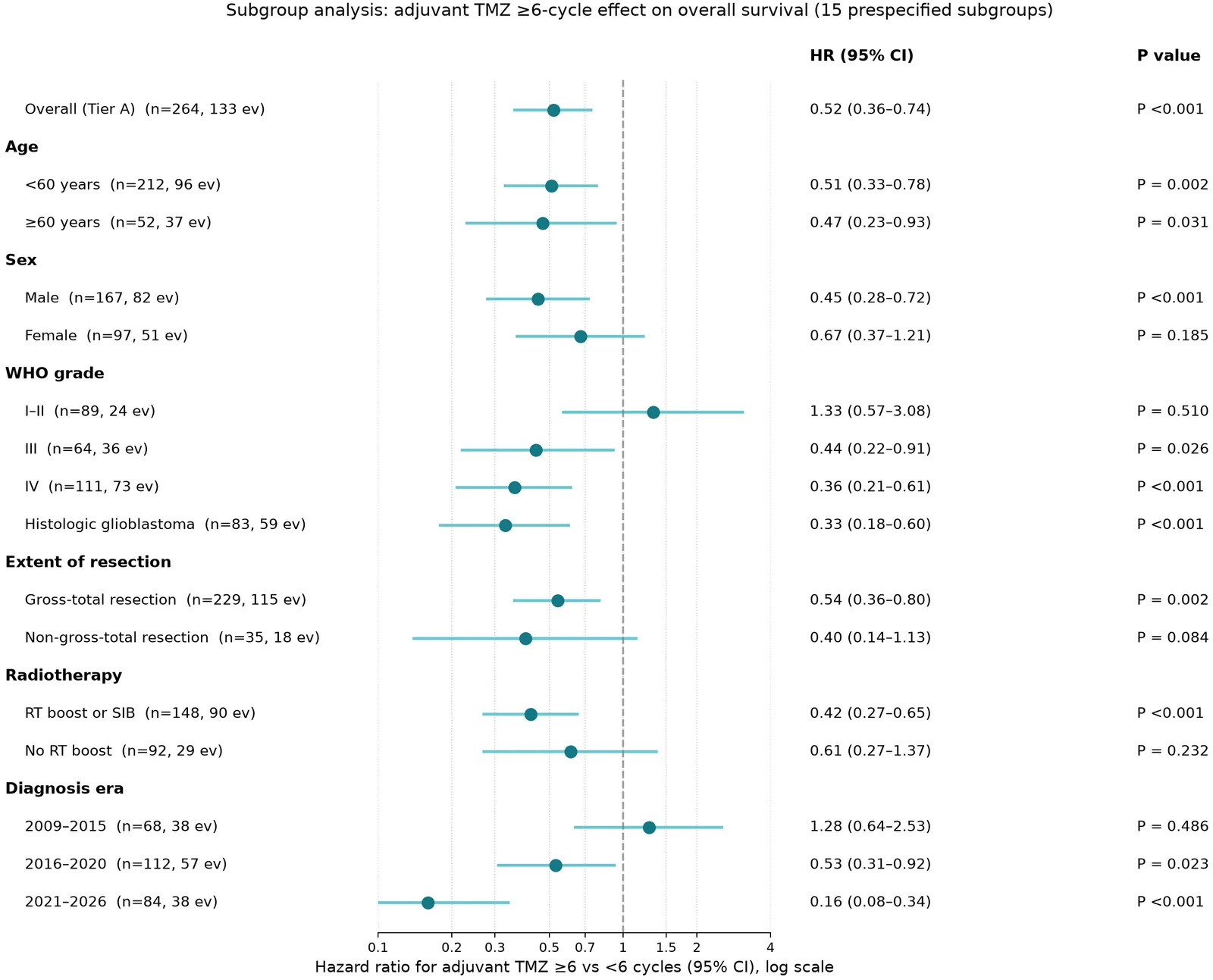
Subgroup analysis of the adjuvant TMZ ≥6-cycle effect on overall survival: 15 prespecified subgroups.

### Cause-specific mortality

Cumulative incidence functions (**Figure 6**) confirmed that tumor-related death dominated overwhelmingly across all grades: the cumulative incidence of non-tumor death remained below 3% at 10 years even in WHO grade IV (only 5 non-tumor deaths were documented among 133 total deaths). Cause-specific stratified Cox regression for tumor-related death (with cause-unknown deaths conservatively attributed to tumor) yielded an HR of 0.45 (95% CI: 0.31–0.67, *P* < 0.001) for adjuvant TMZ ≥6 cycles—indistinguishable from the all-cause OS estimate. The survival benefit thus reflects tumor control, not differential non-tumor mortality.

**Figure 6:**
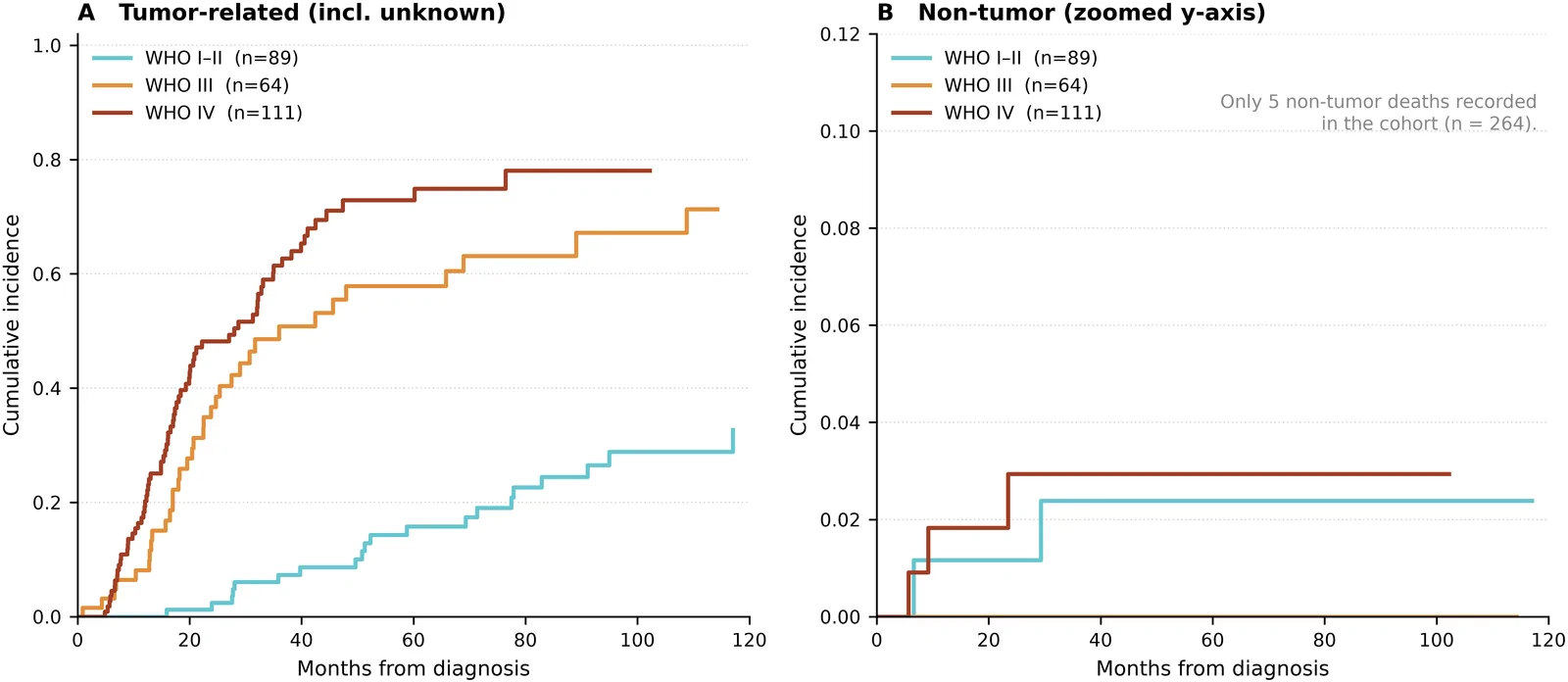
Cumulative incidence of death by cause (Aalen–Johansen), by WHO grade. **(A)** Tumor-related (incl. unknown). **(B)** Non-tumor (zoomed y-axis).

### *E*-value: sensitivity to unmeasured confounding

For the primary stratified Cox HR of 0.46, the *E*-value was 3.79 for the point estimate and 2.33 for the CI limit closer to the null (8). An unmeasured confounder would therefore need to be associated with both TMZ completion and mortality by risk ratios of at least 3.79—beyond age, sex, extent of resection, radiotherapy delivery, RT boost, and targeted therapy—to explain the observed effect away entirely. We interpret the magnitude of this bound in the context of missing molecular annotation in the *Discussion*.

### Sensitivity analyses

Recoding all 34 lost-to-follow-up patients as deaths (a worst-case scenario) preserved the primary conclusion (adjusted HR for TMZ ≥6 = 0.43, 95% CI: 0.30–0.60, *P* < 0.001). The only substantive change was that targeted therapy became marginally significant in the detrimental direction (HR 1.53, 95% CI: 1.01–2.32, *P* = 0.046)—consistent with confounding by indication, since targeted therapy was preferentially deployed in the most refractory patients. Extending the analytic cohort to include tier B patients (*n* = 292) did not alter any conclusion, and excluding the six imputed-day-of-death patients likewise did not.

Three further sensitivity analyses were conducted (**Supplementary Table 5**, **Figure 7**). First, excluding all 45 patients who received targeted (anti-angiogenic/anti-EGFR) therapy left the completion effect essentially unchanged (*n* = 219, 106 deaths; adjusted HR 0.49, 95% CI: 0.32–0.75, *P* = 0.001), confirming that it is not an artifact of targeted therapy use. Second, restricting the model to patients with a documented IDH status (*n* = 184, 89 deaths) recovered a numerically stronger effect (HR 0.35, 95% CI: 0.22–0.57), which was unchanged when IDH mutation status was added as a covariate (HR 0.36, 95% CI: 0.22–0.59, *P* = 0.0001). Third, in the subset with cleanly coded pre-radiotherapy performance status (*n* = 104, 49 deaths, of whom 20 had PS ≥2), adding PS (≥2 vs <2) as a covariate barely moved the point estimate (HR 0.53, 95% CI: 0.28–1.00, *P* = 0.050, vs. 0.52 without PS adjustment); the wider confidence interval reflects the smaller sample rather than attenuation of the effect. These subsets are non-random and are offered as transparency measures rather than as substitutes for the primary model. Descriptively, documented recurrence occurred in 123/264 patients (46.6%) overall—46/108 (42.6%) among ≥6-cycle completers and 77/156 (49.4%) among <6-cycle patients—and rose with grade (WHO II 31.3%, III 50.0%, IV 56.8%).

**Figure 7:**
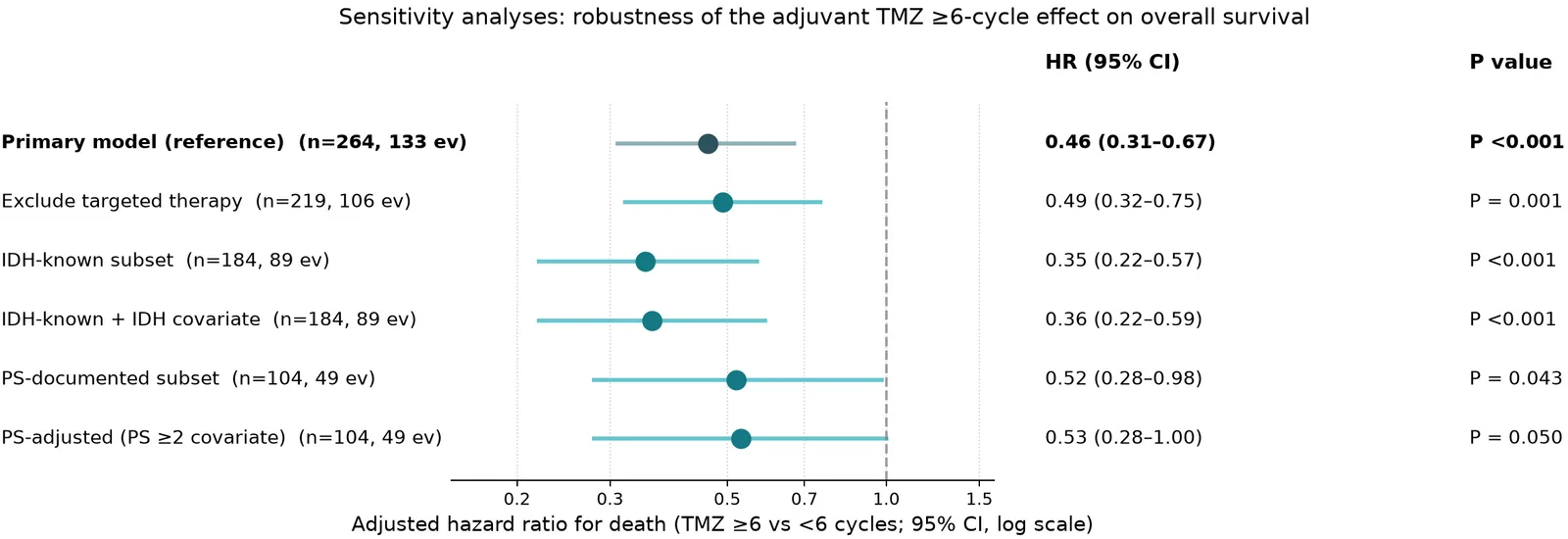
Forest plot of the adjuvant TMZ ≥6-cycle hazard ratio for overall survival across additional sensitivity analyses (exclusion of targeted-therapy recipients; IDH-known subset with and without IDH adjustment; performance status-documented subset with and without PS adjustment), with the primary stratified Cox model as reference.

## Discussion

### Principal findings

In this 17-year single-center real-world Chinese glioma cohort, completing ≥6 cycles of adjuvant temozolomide was the strongest modifiable prognostic factor for OS, halving the adjusted hazard of death (HR 0.46) and translating into a mean gain of 17.0 months at 5 years and 30.3 months at 10 years within grade IV disease. The effect withstood stratification on WHO grade, landmark analyses, inverse-probability-of-treatment weighting, cause-specific modeling, and *E*-value sensitivity analysis. A striking threshold pattern emerged in histologic glioblastoma: 1–5 cycles yielded no measurable advantage over receiving no adjuvant TMZ at all, whereas completing ≥6 cycles conferred a 64% reduction in mortality. The clinical corollary is that the goal of adjuvant therapy is completion of the intended regimen—not merely initiation.

### Placement in the existing literature

The pivotal EORTC/NCIC trial (1) reported a median OS of 14.6 months with concurrent plus adjuvant TMZ; the 5-year update showed 9.8% versus 1.9% survival for TMZ plus radiotherapy versus radiotherapy alone (9). Analyses in Western populations have reproduced OS gains of similar magnitude when patients complete the intended 6 cycles (3, 4), whereas patients who initiate but do not complete adjuvant TMZ occupy an intermediate space. Chinese real-world data—most notably the CGGA registry (10) and various single-center reports—have documented median OS in glioblastoma broadly consistent with Western cohorts once treatment completion is accounted for, although completion rates vary widely (30%–60%). Our overall completion rate of 40.9% (36.0% within grade IV) sits within that range and is consistent with barriers commonly reported in the Chinese context: financial toxicity, family-mediated shared decision-making, and—in the earlier years of the observation window—limited access to bevacizumab and other supportive-care options for TMZ-induced cytopenias. The threshold rather than the dose–response pattern we observed in glioblastoma has been foreshadowed in retrospective analyses of extended TMZ courses (11, 12), although the present analysis is unusual in combining granular per-patient cycle counts with long follow-up. The clinical implication is direct: efforts to improve outcomes in Chinese glioblastoma practice should prioritize adherence-supporting infrastructure—dedicated pharmacy programs, myelotoxicity management pathways, and reimbursement support—with the explicit goal of moving patients from the 1–5-cycle group into the ≥6-cycle group.

A recent report by Sharpe et al. (13) found that adding a stereotactically guided sequential boost to chemoradiotherapy independently improved outcome specifically in MGMT promoter-methylated glioblastoma, while Stupp regimen completion improved OS in both methylation strata, and that older patients undergoing maximal resection and chemoradiation achieved outcomes comparable to younger patients. This bears directly on the adverse aggregate boost/SIB association we report (HR 1.59), which we interpret as confounding by indication: because their boost benefit is conditional on MGMT methylation status, our methylation-agnostic aggregate estimate cannot be read at face value. A structured audit of our records shows a true MGMT promoter-methylation assay result in only 31 of 319 patients, heavily imbalanced (29 methylated vs. 2 unmethylated), with a further ~100 patients having only MGMT immunohistochemistry—an imperfect surrogate for promoter methylation; with just two assay-confirmed unmethylated patients, a methylation-stratified boost analysis of the kind Sharpe/Baskin performed is not estimable in our cohort. Within our grade IV radiotherapy recipients (*n* = 99), boost did not increase 6-cycle completion (36% among boost recipients vs. 44% among non-recipients), and boost-treated grade IV patients had a numerically longer median PFS (15.2 vs 10.2 months); given the adverse aggregate OS association and the absence of assay-based methylation stratification, we attribute this to selection rather than a causal boost effect. Planned molecular re-annotation of archival tissue would allow the methylation-conditional boost hypothesis to be tested directly.

### Methodological considerations

Retrospective evaluation of an intensity-of-treatment exposure such as “completing ≥6 cycles” faces three threats that single-institution reports often address only partially: (1) confounding by indication, because tolerating 6 cycles is itself a marker of favorable biology, performance status, and social support; (2) immortal time bias, because a minimum ~6-month survival is required to be classified in the ≥6-cycle group; and (3) non-proportional hazards over multiyear follow-up. We addressed each explicitly. Confounding was probed by IPTW with excellent postweighting balance (all SMDs < 0.01) and by an *E*-value analysis (the quantitative interpretation in relation to unmeasured molecular confounders is developed below). Immortal time bias was ruled out by four landmark analyses at 3, 6, 9, and 12 months, each of which recovered a highly significant TMZ effect. Non-proportional hazards were handled by stratification on WHO grade (Schoenfeld-guided) and complemented by a time-split model showing that the TMZ benefit is most pronounced in the first 24 months.

### Limitations

Several limitations should be acknowledged transparently. First, and most importantly, molecular annotation (IDH1/2, 1p/19q codeletion, MGMT promoter methylation, ATRX, TERT, H3 K27M) was not systematically available across the observation window, precluding reclassification of the cohort under the 2021 WHO CNS5 scheme. Given the strong prognostic role of these markers—particularly IDH mutation status—residual confounding at the molecular level cannot be excluded. Our *E*-value analysis places a quantitative bound on this concern: an unmeasured confounder would need to be associated with both TMZ completion and mortality by risk ratios of at least 3.79 (2.33 for the CI limit closer to the null) to explain the observed benefit away entirely—a magnitude comparable to the influence of IDH mutation on glioblastoma survival (typical HR 0.3–0.4 for IDH-mutant vs. IDH-wild-type disease (14, 15)). Crucially, however, a confounder must reach this threshold on both legs of the confounder–exposure–outcome triangle simultaneously. Direct comparative data on adjuvant TMZ completion rates between IDH-mutant and IDH-wild-type patients are sparse. Available evidence suggests that IDH-mutant patients enjoy substantially better functional and performance-status outcomes after chemoradiotherapy, with high rates of return to work and preserved KPS (16), and can tolerate extended TMZ courses of up to 12–14 cycles in real-world settings (17). Meta-analytic data confirm that IDH-mutant lower-grade gliomas derive far greater benefit from alkylating chemotherapy than their IDH-wild-type counterparts (OS HR 0.52 vs. 0.87) (18), although this speaks to differential efficacy rather than to differential completion. Notably, in a Chinese cohort of 203 gliomas, IDH mutation did not predict a differential response to concurrent chemoradiotherapy (19). Taken together, the literature supports at most a modest positive association between IDH mutation and TMZ tolerance and completion—clearly below the *E*-value threshold of RR ≥ 3.79 required on the exposure leg. It is therefore unlikely that a fully unmeasured IDH effect could account entirely, rather than partially, for our finding. Retrieval of molecular data from retained pathology blocks is planned for a follow-up analysis and will allow direct empirical testing of this interpretation. Grade-based analysis here is a legacy-histology framework, and our findings should be interpreted as applying to histologically defined glioma rather than to molecularly defined WHO CNS5 entities. A structured audit of existing records shows some IDH determination retrievable in 221 of 319 patients, with an MGMT promoter-methylation assay in only 31, MGMT immunohistochemistry in ~100, 1p/19q in 34, TERT in 4, and CDKN2A/B in 2; on this basis, ~315 of 319 patients would require supplemental testing to meet a CNS5-integrated diagnosis, and prospective testing on retained FFPE blocks is intended to permit a direct, CNS5-anchored re-analysis. Second, the diagnostic edition used for WHO grade evolved across the cohort (2007 → 2016 → 2021 CNS5). We preserved the original diagnostic edition as a covariate rather than reclassifying retrospectively. Third, the extent of resection was classified categorically on early postoperative MRI rather than by volumetric quantification of residual contrast-enhancing tumor. Contemporary consensus criteria (20) operationalize the extent of resection by measured residual contrast-enhancing tumor volume on early postoperative MRI—with “complete resection” defined as no residual contrast enhancement and “near-total resection” as ≤1 cm³ residual—rather than by categorical surgical description. Some patients classified here as “gross total” would likely be reclassified as “near total” under this stricter definition, biasing the estimated non-GTR effect toward the null. This limitation compounds a cohort in which 86.7% of patients were categorized as GTR, leaving only 35 non-GTR patients (18 events)—a distribution that substantially limits statistical power to detect a moderate resection effect. The null was not created by adjustment: the univariate log-rank comparison already yielded *P* = 0.696 (median OS 58.8 vs. 47.4 months), and the hazard ratio remained near unity across sequential models (EOR alone 1.10 → with WHO-grade strata 0.96 → further adding age and sex 0.99 → further adding TMZ ≥6 cycles 1.02; **Supplementary Table 6**). The observed HR range is consistent with that typically reported in retrospective series using categorical MRI-based EOR (approximately 0.8–1.1), rather than with the range from prospective series using volumetric measurement (approximately 0.5–0.7). In an exploratory analysis (**Supplementary Table 6**, Note 4), the direction of the EOR effect was compatible with an EOR × adjuvant therapy interaction (non-GTR HR 0.74 within TMZ ≥6-cycle completers vs. 1.39 within TMZ <6-cycle patients), although power was insufficient to establish this formally (interaction *P* = 0.19). Fourth, cause of death was determined from family reports or medical records if a patient dies at a hospital without autopsy confirmation; however, tumor-related death dominated the cause distribution to such an extent that this uncertainty is unlikely to have materially affected the results, as demonstrated by the near-identical HRs for all-cause OS and cause-specific mortality. Fifth, recurrence was defined by any imaging finding consistent with recurrence rather than by central RANO-based imaging review (21); PFS estimates should therefore be interpreted cautiously. Sixth, the cohort is single center in southern China; generalizability to other Chinese regions and to non-Chinese populations requires validation. Seventh, 34 patients (10.7%) were lost to follow-up; a sensitivity analysis re-coding these patients as deaths did not alter the primary conclusion. Eighth, baseline functional/performance status (KPS/ECOG) was recorded inconsistently across the 17-year window and was therefore not included as a covariate in the primary model; residual confounding by baseline functional status cannot be excluded, and part of the observed association may reflect the better baseline condition of patients able to complete 6 cycles. A performance status-adjusted sensitivity analysis in the subset with cleanly coded pre-radiotherapy status (*n* = 104) left the completion effect essentially unchanged (HR 0.53 vs 0.52; see *Results, Sensitivity analyses*). Ninth, post-progression management (re-operation, re-irradiation, temozolomide re-challenge, anti-angiogenic agents, and best supportive care) was heterogeneous and not protocolized, and re-operation and re-irradiation at recurrence were not systematically coded; because salvage therapy is a post-exposure (downstream) variable, adjusting for it in the primary model would risk mediator/over-adjustment bias, so it is described descriptively (*Results, Sensitivity analyses*) rather than entered into the multivariable model.

### Clinical implications

Our findings support two operational priorities for Chinese neuro-oncology practice. First, completion of the intended 6 cycles of adjuvant TMZ should be treated as an explicit quality-of-care target, tracked as a program-level metric rather than assumed once the regimen is initiated. Second, patient-selection biomarkers that identify who is most likely to tolerate and benefit from a full course would substantially strengthen the value of the regimen; completing molecular annotation on legacy pathology material is a natural extension of the present work.

## Conclusions

In this rigorously analyzed 17-year Chinese real-world glioma cohort, completing ≥6 cycles of adjuvant temozolomide emerged as the single strongest modifiable prognostic factor for OS—translating into a mean of 17.0 additional months of 5-year survival in glioblastoma and remaining robust across multiple bias-mitigating analytic frameworks. Ensuring—not merely prescribing—adjuvant temozolomide completion warrants elevation to a formal quality-of-care standard in Chinese neuro-oncology practice.

## Data Availability

The original contributions presented in the study are included in the article/**Supplementary Material**. Further inquiries can be directed to the corresponding authors.
