# Supplementary material for: Completion of ≥6 cycles of adjuvant temozolomide is the strongest modifiable prognostic factor in glioblastoma: a 17-year real-world cohort of 319 glioma patients from a single Chinese center
Source: Front Oncol. 2026 Sep 16;16:1933534. doi: 10.3389/fonc.2026.1933534 (PMC13623740; doi:10.3389/fonc.2026.1933534)
Supplement: Supplementary file 1 [file Table1.docx]

**Supplementary Table S1. Per-variable completeness accounting in the full registered cohort (n = 319) and the primary analysis Tier A cohort (n = 264).**

| **Category** | **Variable** | **Description** | **Missing, full cohort (n = 319)** | **Missing, Tier A (n = 264)** |
| --- | --- | --- | --- | --- |
| **Demographics** | sex | Male / Female | 0 (0.0%) | 0 (0.0%) |
|  | age_years | Age at diagnosis | 0 (0.0%) | 0 (0.0%) |
|  | age_group | <18 / 18-39 / 40-59 / >=60 | 0 (0.0%) | 0 (0.0%) |
| **Diagnosis & timing** | dx_date | Date of diagnosis | 1 (0.3%) | 0 (0.0%) |
|  | dx_era | Diagnosis era: 2009–2015 / 2016–2020 / 2021–2026 | 1 (0.3%) | 0 (0.0%) |
|  | who_classification_edition | WHO CNS classification edition assigned by year of diagnosis: 2007 / 2016 / 2021 (CNS5) | 0 (0.0%) | 0 (0.0%) |
|  | surgery_date | Date of first surgery | 1 (0.3%) | 0 (0.0%) |
|  | last_followup_date | Date of most recent successful follow-up contact | 0 (0.0%) | 0 (0.0%) |
|  | death_date | Date of death (day imputed as the 15th of the reported month when day was missing) | 165 (51.7%) | 136 (51.5%) |
|  | survival_status | Dead / Alive / Lost | 0 (0.0%) | 0 (0.0%) |
|  | event_death | Death event indicator (Alive / Lost = 0) | 0 (0.0%) | 0 (0.0%) |
|  | os_months | OS (months) = (date of death or last follow-up − date of diagnosis) / 30.4375 | 1 (0.3%) | 0 (0.0%) |
| **Tumor characteristics** | histology | Histologic subtype, categorised from the original pathology diagnosis | 0 (0.0%) | 0 (0.0%) |
|  | who_grade | Harmonised WHO grade (upgrade at recurrence coded as X_mixed) | 2 (0.6%) | 0 (0.0%) |
|  | who_grade_primary | Primary WHO grade after separating mixed-grade cases | 2 (0.6%) | 0 (0.0%) |
|  | who_grade_original | Original pathology-grade text as recorded (not harmonised) | 2 (0.6%) | 0 (0.0%) |
|  | tumor_location | Anatomical location of the tumor | 0 (0.0%) | 0 (0.0%) |
|  | tumor_side | Left / Right / Bilateral / Unspecified | 0 (0.0%) | 0 (0.0%) |
|  | tumor_max_dim_mm | Maximum tumor dimension on preoperative imaging (mm) | 64 (20.1%) | 29 (11.0%) |
|  | extent_of_resection | Extent-of-resection category | 0 (0.0%) | 0 (0.0%) |
| **Radiotherapy** | rt_received | Receipt of radiotherapy (=1 if a dose was resolvable or receipt explicitly documented) | 0 (0.0%) | 0 (0.0%) |
|  | rt_start_date | Date radiotherapy started | 39 (12.2%) | 22 (8.3%) |
|  | rt_end_date | Date radiotherapy ended | 47 (14.7%) | 27 (10.2%) |
|  | surg_to_rt_days | Interval from surgery to start of radiotherapy (days) | 35 (11.0%) | 21 (8.0%) |
|  | rt_total_dose_gy | Total prescribed dose (Gy); maximum across multiple PTVs; sum of A+B for boost regimens | 42 (13.2%) | 24 (9.1%) |
|  | rt_total_fractions | Total number of fractions; sum of A+B for boost regimens; maximum across multiple PTVs | 42 (13.2%) | 24 (9.1%) |
|  | rt_dose_per_fraction_gy | Dose per fraction (Gy/fx) = rt_total_dose_gy / rt_primary_fractions; taken from the primary phase in A+B boost regimens | 42 (13.2%) | 24 (9.1%) |
|  | rt_primary_dose_gy | Prescribed dose of the primary (phase 1 / low-dose) PTV | 42 (13.2%) | 24 (9.1%) |
|  | rt_boost_present | Presence of simultaneous integrated or sequential boost | 0 (0.0%) | 0 (0.0%) |
|  | rt_boost_dose_gy | Dose delivered to the boost volume (Gy); B component for A+B; (max − min) across multiple PTVs | 145 (45.5%) | 116 (43.9%) |
| **Systemic therapy** | concurrent_tmz | Concurrent temozolomide administered during radiotherapy | 0 (0.0%) | 0 (0.0%) |
|  | adjuvant_tmz | Adjuvant temozolomide administered after radiotherapy | 0 (0.0%) | 0 (0.0%) |
|  | adjuvant_tmz_cycles | Number of adjuvant temozolomide cycles | 9 (2.8%) | 5 (1.9%) |
|  | adjuvant_tmz_ge6cycles | Completion of ≥6 cycles of adjuvant temozolomide | 9 (2.8%) | 5 (1.9%) |
|  | stupp_like_regimen | Stupp-like regimen indicator (radiotherapy + concurrent TMZ + any adjuvant TMZ) | 0 (0.0%) | 0 (0.0%) |
|  | targeted_therapy | Receipt of any targeted therapy | 0 (0.0%) | 0 (0.0%) |
| **Outcome / follow-up** | pfs_months | PFS (months) = (date of first recurrence, death, or last follow-up − date of diagnosis) / 30.4375 | 1 (0.3%) | 0 (0.0%) |
|  | event_progression | Progression event indicator (recurrence or death = 1) | 0 (0.0%) | 0 (0.0%) |
|  | recurrence | Any documented evidence of recurrence (imaging suspicious for recurrence counted as an event) | 0 (0.0%) | 0 (0.0%) |
|  | first_recurrence_date | Date of first recurrence | 164 (51.4%) | 141 (53.4%) |
|  | metastasis | Any documented metastasis | 0 (0.0%) | 0 (0.0%) |
|  | death_cause_category | Cause-of-death category: Tumor-related / Infection / Cardio-cerebrovascular / Other / Unknown (derived from family report; reliability limited) | 157 (49.2%) | 129 (48.9%) |

*Notes: (1) Tier A gating fields have 0% completeness by construction; these are WHO grade, diagnosis date, survival status, overall survival months, extent of resection, and (in radiotherapy recipients) total radiotherapy dose. (2) death_date completeness (51.7%) is not a data-quality issue; it reflects the 165 patients still alive or lost to follow-up at the database lock on 30 June 2026, for whom overall survival is calculated to last documented contact. (3) first_recurrence_date completeness (51.4%) reflects the fraction of patients without documented recurrence, either because none had occurred or because recurrence dating was ambiguous; progression-free survival analyses censor these patients at last follow-up. (4) death_cause_category completeness (49.2%) likewise reflects patients not yet deceased plus a small number of decedents for whom family reports did not yield a categorical cause. (5) tumor_max_dim_mm (20.1% overall / 11.0% Tier A) is the largest completeness value for a variable used descriptively in Table 1; a sensitivity analysis that excluded this variable did not change the multivariable results. (6) rt_boost_dose_gy (45.5% overall / 43.9% Tier A) is missing structurally, because the field is defined only when rt_boost_present = 1; restricted to the 148 Tier A patients who received a boost or SIB, completeness is 8.1%. (7) No imputation was performed for the primary analysis.*
