# Supplementary material for: Completion of ≥6 cycles of adjuvant temozolomide is the strongest modifiable prognostic factor in glioblastoma: a 17-year real-world cohort of 319 glioma patients from a single Chinese center
Source: Front Oncol. 2026 Sep 16;16:1933534. doi: 10.3389/fonc.2026.1933534 (PMC13623740; doi:10.3389/fonc.2026.1933534)
Supplement: Supplementary file 2 [file Table2.docx]

# Supplementary Table S2. Multi-timepoint landmark analysis of the adjuvant TMZ ≥6-cycle effect

| **Timepoint (months)** | **Patients still at risk** | **Events**  **(deaths)** | **Adjusted HR (TMZ ≥6 vs <6)** | **95% CI** | **P value** |
| --- | --- | --- | --- | --- | --- |
| 3 | 263 | 132 | 0.44 | 0.30–0.65 | <0.001 |
| 6 | 251 | 126 | 0.47 | 0.32–0.70 | <0.001 |
| 9 | 234 | 112 | 0.54 | 0.36–0.82 | 0.003 |
| 12 | 220 | 104 | 0.60 | 0.40–0.92 | 0.019 |

## *Notes*

1. *HRs are from the multivariable stratified Cox regression (strata = WHO grade), adjusting for age ≥60 years, male sex, non-gross-total resection, no radiotherapy, radiotherapy boost/SIB, and receipt of targeted therapy — the same covariates as the primary model.*
2. *The 3-month landmark HR (0.44) is nearly identical to the unlanded primary-model estimate (0.46), demonstrating that patients who go on to complete ≥6 cycles do not differ from those who complete <6 cycles in early mortality risk that could be attributed to “surviving long enough” to receive more cycles.*
3. *Attenuation of the effect at the 12-month landmark (HR 0.60) is expected: patients still alive at 12 months are enriched for those already completing (or nearly completing) their intended course, so the exposure contrast is reduced.*
