# Supplementary material for: Completion of ≥6 cycles of adjuvant temozolomide is the strongest modifiable prognostic factor in glioblastoma: a 17-year real-world cohort of 319 glioma patients from a single Chinese center
Source: Front Oncol. 2026 Sep 16;16:1933534. doi: 10.3389/fonc.2026.1933534 (PMC13623740; doi:10.3389/fonc.2026.1933534)
Supplement: Supplementary file 3 [file Table3.docx]

**Supplementary Table S3. Restricted mean survival time (RMST) at 60 and 120 months, by WHO grade and adjuvant temozolomide exposure (n = 264).**

| **Stratum and horizon** | **n (≥6)** | **n (<6)** | **RMST ≥6** | **RMST <6** | **ΔRMST** | **95% CI for ΔRMST** | **P value** |
| --- | --- | --- | --- | --- | --- | --- | --- |
| **WHO grade I–II** |  |  |  |  |  |  |  |
| RMST at 60 months (months) | 41 | 48 | 55.1 | 56.4 | −1.3 | −6.4 to +3.9 | 0.606 |
| RMST at 120 months (months) | 41 | 48 | 97.2 | 102.0 | −4.7 | −20.6 to +11.3 | 0.564 |
| **WHO grade III** |  |  |  |  |  |  |  |
| RMST at 60 months (months) | 27 | 37 | 43.6 | 34.2 | +9.3 | −0.4 to +20.2 | 0.081 |
| RMST at 120 months (months) | 27 | 37 | 72.6 | 50.4 | +22.2 | −2.7 to +45.9 | 0.078 |
| **WHO grade IV** |  |  |  |  |  |  |  |
| RMST at 60 months (months) | 40 | 71 | 41.1 | 24.1 | +17.0 | +9.2 to +24.7 | <0.001 |
| RMST at 120 months (months) | 40 | 71 | 61.5 | 31.2 | +30.3 | +11.8 to +48.8 | 0.001 |

*Abbreviations: CI, confidence interval; ΔRMST, difference in restricted mean survival time (≥6 cycles minus <6 cycles); RMST, restricted mean survival time; TMZ, temozolomide.*

*Notes: RMST is estimated non-parametrically as the area under the Kaplan–Meier survival curve up to the specified horizon (60 or 120 months). Confidence intervals for ΔRMST are derived from 500-replicate bootstrap resampling; P values use a normal approximation with the bootstrap-derived standard error. RMST was chosen as a hazard-ratio-independent effect measure appropriate for comparisons in which the proportional-hazards assumption is violated (Uno et al., 2014). Interpretation: ΔRMST is the mean number of additional months of survival within the specified horizon attributable to completion of ≥6 versus <6 cycles of adjuvant temozolomide. Within WHO grade IV disease, completion of ≥6 cycles corresponds to 17.0 additional months of survival over 5 years and 30.3 additional months over 10 years; the corresponding grade III differences trend in the same direction but do not reach statistical significance. WHO grade I–II shows no meaningful ΔRMST, consistent with adjuvant TMZ not being standard of care in this stratum.*
