# Supplementary material for: Completion of ≥6 cycles of adjuvant temozolomide is the strongest modifiable prognostic factor in glioblastoma: a 17-year real-world cohort of 319 glioma patients from a single Chinese center
Source: Front Oncol. 2026 Sep 16;16:1933534. doi: 10.3389/fonc.2026.1933534 (PMC13623740; doi:10.3389/fonc.2026.1933534)
Supplement: Supplementary file 4 [file Table4.docx]

# Supplementary Table S4. Subgroup analysis of the adjuvant TMZ ≥6-cycle effect on overall survival

| **Subgroup** | **n** | **Events** | **HR** | **95% CI** | **P value** |
| --- | --- | --- | --- | --- | --- |
| Overall (Tier A) | 264 | 133 | 0.52 | 0.36–0.74 | <0.001 |
| Age |  |  |  |  |  |
| <60 years | 212 | 96 | 0.51 | 0.33–0.78 | 0.002 |
| ≥60 years | 52 | 37 | 0.47 | 0.23–0.93 | 0.031 |
| Sex |  |  |  |  |  |
| Male | 167 | 82 | 0.45 | 0.28–0.72 | <0.001 |
| Female | 97 | 51 | 0.67 | 0.37–1.21 | 0.185 |
| WHO grade |  |  |  |  |  |
| I–II | 89 | 24 | 1.33 | 0.57–3.08 | 0.510 |
| III | 64 | 36 | 0.44 | 0.22–0.91 | 0.026 |
| IV | 111 | 73 | 0.36 | 0.21–0.61 | <0.001 |
| Histologic glioblastoma | 83 | 59 | 0.33 | 0.18–0.60 | <0.001 |
| Extent of resection |  |  |  |  |  |
| Gross-total resection | 229 | 115 | 0.54 | 0.36–0.80 | 0.002 |
| Non-gross-total resection | 35 | 18 | 0.40 | 0.14–1.13 | 0.084 |
| Radiotherapy |  |  |  |  |  |
| RT boost or SIB | 148 | 90 | 0.42 | 0.27–0.65 | <0.001 |
| No RT boost | 92 | 29 | 0.61 | 0.27–1.37 | 0.232 |
| Diagnosis era |  |  |  |  |  |
| 2009–2015 | 68 | 38 | 1.28 | 0.64–2.53 | 0.486 |
| 2016–2020 | 112 | 57 | 0.53 | 0.31–0.92 | 0.023 |
| 2021–2026 | 84 | 38 | 0.16 | 0.08–0.34 | <0.001 |
