# Supplementary material for: Completion of ≥6 cycles of adjuvant temozolomide is the strongest modifiable prognostic factor in glioblastoma: a 17-year real-world cohort of 319 glioma patients from a single Chinese center
Source: Front Oncol. 2026 Sep 16;16:1933534. doi: 10.3389/fonc.2026.1933534 (PMC13623740; doi:10.3389/fonc.2026.1933534)
Supplement: Supplementary file 5 [file Table5.docx]

# Supplementary Table S5. Additional sensitivity analyses of the adjuvant TMZ ≥6-cycle effect on overall survival

| **Scenario** | **Cohort** | **N (deaths)** | **Adjusted HR, TMZ ≥6 vs <6** | **Interpretation** |
| --- | --- | --- | --- | --- |
| Primary model (reference) | All Tier A | 264 (133) | 0.46 (95% CI 0.31–0.67), *P* < 0.001 | Reference estimate for completing ≥6 adjuvant TMZ cycles. |
| Exclude targeted-therapy recipients | Tier A minus 45 targeted-therapy patients | 219 (106) | 0.49 (95% CI 0.32–0.75), *P* = 0.001 | Removing all anti-angiogenic/anti-EGFR recipients leaves the effect essentially unchanged; not an artefact of targeted therapy. |
| IDH-known subset | Tier A with documented IDH status | 184 (89) | 0.35 (95% CI 0.22–0.57), *P* < 0.001 | In the molecularly characterised subset the effect persists and is numerically stronger. |
| IDH-known subset + IDH covariate | Tier A with documented IDH status | 184 (89) | 0.36 (95% CI 0.22–0.59), *P* = 0.0001 | Adjusting for IDH mutation status does not attenuate the effect. |
| Performance-status–documented subset | Tier A with cleanly coded pre-RT PS | 104 (49) | 0.52 (95% CI 0.28–0.98), *P* = 0.043 | Effect preserved in the subset with recorded performance status. |
| PS-adjusted (PS ≥2 vs <2 covariate) | Same subset (20 with PS ≥2) | 104 (49) | 0.53 (95% CI 0.28–1.00), *P* = 0.050 | Adding baseline PS barely moves the point estimate (0.53 vs 0.52); CI widens with the smaller sample. |

***Note (descriptive, not modelled).*** *Documented recurrence occurred in 123/264 patients (46.6%) overall — 46/108 (42.6%) among ≥6-cycle completers and 77/156 (49.4%) among <6-cycle patients — and rose with grade (WHO II 31.3%, III 50.0%, IV 56.8%). Salvage systemic therapy (anti-angiogenic/anti-EGFR) was recorded in 45 patients; re-operation and re-irradiation were not systematically coded. These non-random subsets are offered as transparency measures rather than as substitutes for the primary model.*
