# Supplementary material for: Completion of ≥6 cycles of adjuvant temozolomide is the strongest modifiable prognostic factor in glioblastoma: a 17-year real-world cohort of 319 glioma patients from a single Chinese center
Source: Front Oncol. 2026 Sep 16;16:1933534. doi: 10.3389/fonc.2026.1933534 (PMC13623740; doi:10.3389/fonc.2026.1933534)
Supplement: Supplementary file 6 [file Table6.docx]

# Supplementary Table S6. Sequential Cox regression of extent of resection (non-GTR vs GTR)

| **Model** | **Covariates in model** | **Stratified on** | **HR (non-GTR vs GTR)** | **95% CI** | **P value** |
| --- | --- | --- | --- | --- | --- |
| A | EOR alone (unadjusted) | none | 1.10 | 0.67–1.82 | 0.696 |
| B | + WHO grade | strata = WHO grade | 0.96 | 0.58–1.58 | 0.860 |
| C | + age ≥60 + sex | strata = WHO grade | 0.99 | 0.60–1.63 | 0.954 |
| D | + adjuvant TMZ ≥6 cycles | strata = WHO grade | 1.02 | 0.62–1.69 | 0.932 |
| Primary model (Fig 4 / Table 2) | age, sex, RT boost/SIB, no RT, TMZ ≥6, targeted therapy | strata = WHO grade | 1.04 | 0.63–1.72 | 0.888 |

## *Notes*

1. ***The point estimate never leaves the neighborhood of 1.0.*** *The univariate log-rank comparison (before any Cox modeling) also failed to reject the null: median OS was 58.8 months for GTR versus 47.4 months for non-GTR (log-rank P = 0.696).*
2. ***The null effect is not driven by over-adjustment.*** *If the primary Cox model were "absorbing" a real EOR signal into other covariates, the HR would drift systematically toward 1 as covariates were added. Instead the HR moves within a narrow band (0.96–1.10) that already straddles 1.*
3. *Two structural features of the cohort explain this finding. First, 86.7% of the cohort (229/264) was classified as GTR — an unusually high rate compared with prospective GBM series (e.g. 39% in the pivotal EORTC 26981/Stupp trial). Second, EOR was determined by categorical reading of early postoperative MRI (48–72 h) rather than by volumetric quantification of residual enhancing tumor. Contemporary consensus criteria [Karschnia 2021, ref [20] in main text] operationalize extent of resection by measured residual contrast-enhancing tumor volume on early postoperative MRI — with "complete resection" defined as no residual contrast enhancement and "near-total resection" as ≤ 1 cm³ residual — rather than by categorical surgical description. Under this stricter definition, some patients classified here as "gross total" would likely be reclassified as "near total", biasing the effect estimate toward the null. The observed HR range is consistent with that typically reported in retrospective series using categorical MRI-based EOR (approximately 0.8–1.1), rather than with the range from prospective series using volumetric measurement (approximately 0.5–0.7).*
4. *Exploratory EOR × TMZ interaction. Within TMZ ≥6-cycle completers, the adjusted HR for non-GTR was 0.74 (95% CI 0.28–1.92); within TMZ <6-cycle patients, it was 1.39 (0.77–2.52). Directionally, this is compatible with the "EOR × adjuvant therapy" synergy described in the Stummer 5-ALA and Sanai literature, in which surgical benefit is amplified when adjuvant therapy is completed. The formal interaction test did not reach significance (likelihood-ratio P = 0.19), consistent with limited power in the small non-GTR subgroup (n = 35, 18 events).*
